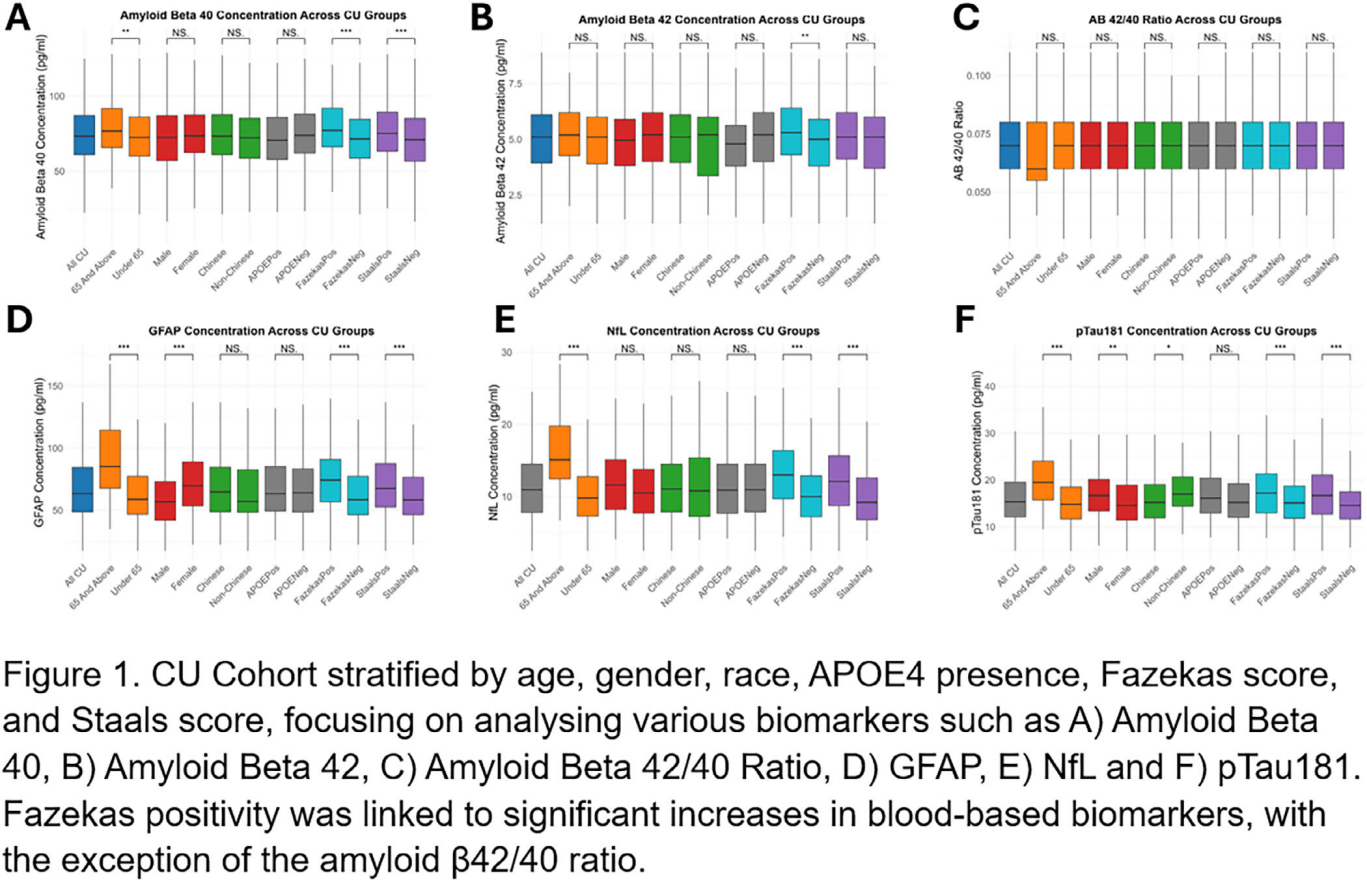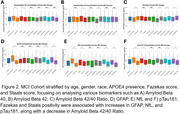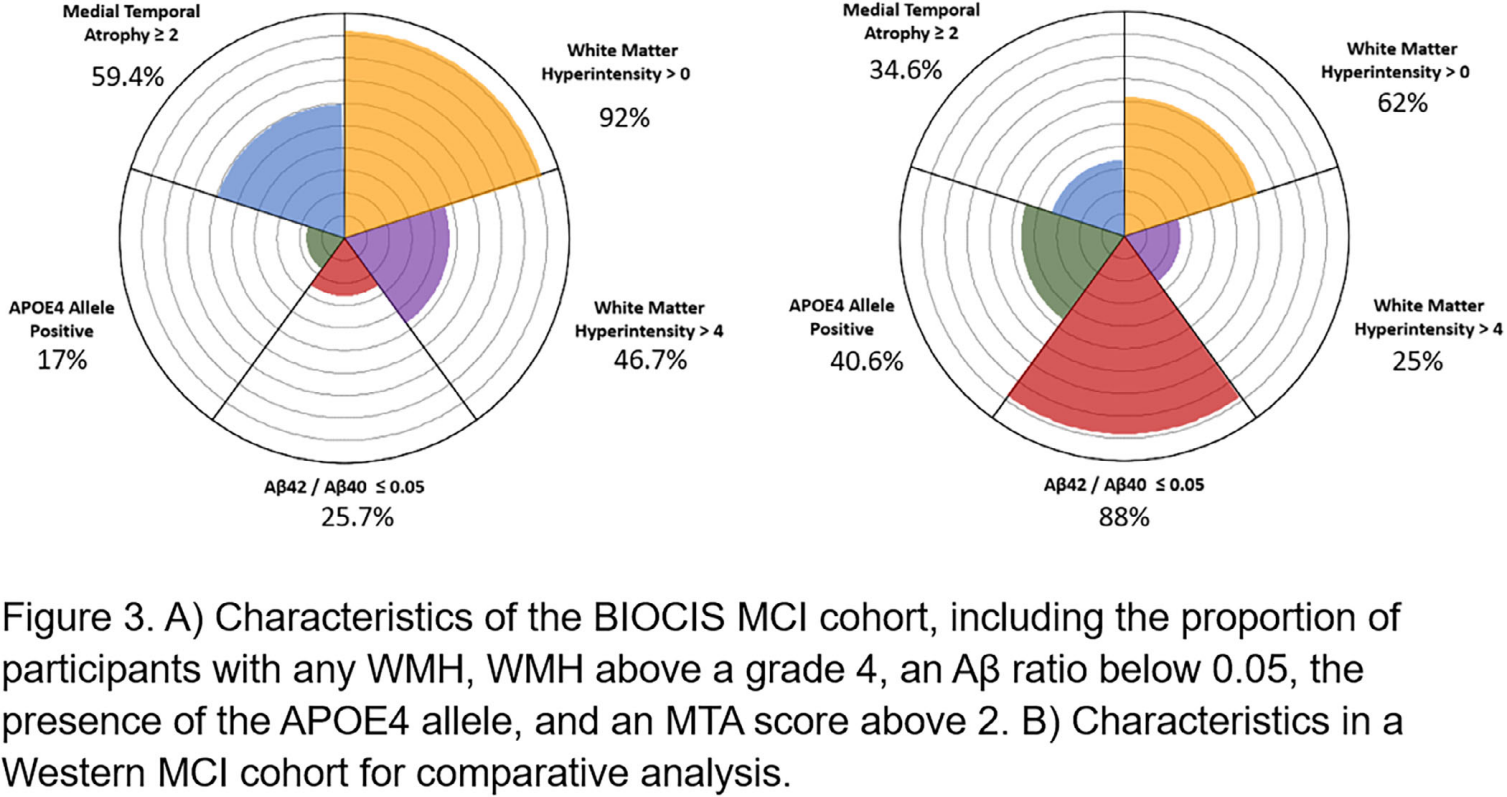# The influence of demographic, genetic and neurodegenerative variations on biomarker profiles in a Southeast Asian community cohort

**DOI:** 10.1002/alz70856_100105

**Published:** 2025-12-24

**Authors:** Jacklyn Leonardo, Gurveen Kaur Sandhu, Ashwati Vipin, Bocheng Qiu, Rasyiqah Binte Shaik Mohamed Salim, Nagaendran Kandiah

**Affiliations:** ^1^ Dementia Research Centre (Singapore), Lee Kong Chian School of Medicine, Nanyang Technological University, Singapore, Singapore; ^2^ Lee Kong Chian School of Medicine, Nanyang Technological University, Singapore, Singapore; ^3^ Dementia Research Centre (Singapore), Lee Kong Chian School of Medicine, Nanyang Technological University, Singapore 308232, Singapore, Singapore; ^4^ Neuroscience and Mental Health Programme, Lee Kong Chian School of Medicine, Nanyang Technological University, Singapore, Singapore; ^5^ National Healthcare Group, Singapore, Singapore

## Abstract

**Background:**

Dementia research is largely centred on Western populations, with limited focus on Southeast Asia despite its unique ethnic diversity. Singapore, home to Chinese, Malay, and Indian populations, offers a rare opportunity to explore how shared genetics but differing lifestyles and environments influence dementia risk. To date, no large‐scale study has examined how demographic factors, genetic variations, and cerebral white matter changes affect blood‐based biomarkers in this region. Addressing these gaps is critical for understanding biomarker variability and its implications for dementia diagnosis and management in Southeast Asia.

**Method:**

This study analysed 1,646 participants in the Biomarker and Cognition Study Singapore (BIOCIS), a longitudinal study at the Dementia Research Centre, Singapore. Participants were classified as Cognitively Unimpaired (CU) or with Mild Cognitive Impairment (MCI) and further stratified by age, gender, race, APOE ε4 carrier status, Fazekas score (≥2), and Staals score (≥1). Plasma biomarkers, including Aβ40, Aβ42, Aβ42/40 ratio, GFAP, pTau181, and NfL, were compared between groups using independent samples t‐tests. Additionally, comparisons were made between the BIOCIS MCI cohort and Western MCI cohorts based on published trends in the field.

**Result:**

Fazekas positivity was associated with significant differences in blood‐based biomarkers, except for the Aβ42/40 ratio, in the CU cohort. In the MCI cohort, vascular burden, indicated by Fazekas and Staals positivity, was linked to increased levels of GFAP(Fazekas and Staals *p* <0.0001), pTau181(Fazekas *p* = 0.004, Staals *p* <0.001), and NfL(Fazekas and Staals *p* <0.0001) levels. The BIOCIS MCI cohort shows lower amyloid burden(25.7% vs. 88%) and lower APOE4 positivity(17% vs. 40.6%) but higher WMH prevalence(92% vs. 62%) compared to Western cohorts.

**Conclusion:**

Our study indicates that Fazekas positivity influences biomarker profiles in Southeast Asians, contributing to higher levels of amyloid burden, GFAP, NfL, and pTau181, highlighting the role of vascular factors in early brain changes linked to dementia risk. Within the MCI group, vascular burden persists in influencing the profiles, suggesting a carryover of burden. The distinct biomarker profiles in Southeast Asia, compared to the Western cohorts, highlights the need for region‐specific research to improve understanding and care. Singapore's unique combination of shared genetics and diverse lifestyles makes it a critical location for studying these effects.